# In the Labyrinth of Dietary Patterns and Well-Being—When Eating Healthy Is Not Enough to Be Well

**DOI:** 10.3390/ijerph19031259

**Published:** 2022-01-23

**Authors:** Renata Nestorowicz, Ewa Jerzyk, Anna Rogala

**Affiliations:** Institute of Marketing, Department of Marketing Strategies, Poznań University of Economics and Business (PUEB), Niepodległości Av. 10, 61-875 Poznań, Poland; renata.nestorowicz@ue.poznan.pl (R.N.); ewa.jerzyk@ue.poznan.pl (E.J.)

**Keywords:** food consumption, well-being, dietary patterns, sustainable consumption, consumer behaviour, food well-being, subjective well-being

## Abstract

This paper aims to identify the relation between food consumption and well-being, and the level of well-being depending on a diet followed. Moreover, we analyze whether people driven by single motives, such as the health, pleasure or social dimension of food declare the lower or higher level of well-being than those motivated by a larger number of factors. The survey was conducted online (CAWI, *n* = 1067). The following scales were used: Satisfaction With Life Scale (SWLS), Satisfaction with Food-related Life Scale (SWFL), Health Taste Attitude Scales (HTAS) and Social Dimension of Food Meaning. The data analysis was carried out with the application of one-way analysis of variance (ANOVA), partial eta squared, a t-Student’s test, the Hochberg test, the Games-Howell test, and Pearson’s correlation. Levels of Subjective Well-Being (SWB) and Food Well-Being (FWB) are strongly correlated with consumers’ dietary pattern. The frequency of consumption of organic food and following vegan, low salt, and low sugar diets leads to higher levels of SWB, while FWB is additionally affected by the regular consumption of low-fat products and foods that improve one’s mood. The level of well-being is linked with the motivation to follow specific diets and attentiveness related to dietary patterns. People paying attention to the health aspects, pleasure and social dimension of food meaning show higher level of FWB than people focusing exclusively on health aspects.

## 1. Introduction

In well-being studies, there are two different approaches to analysing the contribution of food consumption to the individual’s life satisfaction. In subjective well-being (SWB) studies, food consumption is considered to be one of the factors contributing to the overall quality of life satisfaction e.g., [1,2,3], whereas food well-being (FWB) studies provide an insight into the perception of the quality of life resulting from food consumption in general, e.g., [4,5,6,7,8,9,10,11]. Subjective well-being is understood as the subjective evaluation of one’s life from both an affective and a cognitive perspective [12]. On the other hand, food well-being is defined as the “positive psychological, physical, emotional, and social relationship with food at both the individual and societal levels” [4]. The findings of numerous studies prove that there is a link between food consumption and the well-being of an individual e.g., [7,13,14,15,16]. Moreover, a diet influences FWB and it is also a source of pleasure-related emotions [17].

This paper aimed to examine the relations between food consumption and subjective and food well-being. We undertook identifying consumers’ well-being from the perspective of consumption of specific food categories, such as organic, ethical, convenient, sugar-free, gluten-free, and fat-reduced food, and discover which dietary patterns lead to increasing FWB and SWB. We also examined relations between FWB and SWB among Polish consumers, referring the obtained results to the findings of international studies. So far, only a few studies have been carried out to discover the impact of diets that reduce or exclude the consumption of specific product categories on one’s well-being. Thus, our study fills this gap as it concerns dietary patterns and well-being.

At present, consumers show a wide variety of attitudes to diets—from the over-consumption of processed food, through a moderate and balanced diet, to reducing the consumption of specific groups of products or ingredients. At the same time, dietary guidelines worldwide include similar recommendations concerning healthy eating: one should eat diverse grocery products in proper proportions, more fruit, vegetables and whole-grain foods, less sugar, fat and salt [18]. In recent years, some new guidelines have been added to this list. They are related to the influence of a diet on the environment and to the adjustment of dietary patterns to more sustainable consumption [19]. The production of the food of animal origin, such as meat and dairy products, is identified as the key factor in soil degradation, loss of diversity and climate change. According to Vieux et al. [20], the adoption of a healthy diet, which reduces greenhouse gas emissions by thirty per cent, would require replacing of fifty per cent of the current food consumption with other food products. We are observing the fast growth of the segment of ethical consumers, who make their dietary decisions motivated by environmental considerations (cruelty and exploitation of animals, greenhouse gases) and by problems of social inequality when it comes to access to environmental resources by the present and future generations [21]. A vegetarian diet is becoming increasingly popular in Western societies, where it is preferred by the estimated number of one to nine per cent of consumers [22,23].

There is also a phenomenon of food fashions in the market, including diets based on so-called superfoods, such as chia seeds, goji berry, spirulina, linseed, walnut or kale. This phenomenon is observed among consumers following a gluten-free diet, who, having no diagnosed coeliac disease or gluten sensitivity, choose this diet despite it being more expensive and less nutritious. It is estimated that only about one per cent of the population suffer from coeliac disease and from three to six per cent are gluten sensitive. Still, as many as 20 per cent of the population choose a gluten-free diet without any medical indications [24]. Moreover, the market offers a wide range of organic, “free from” products, weight management articles, and fortified and functional products, which constitute the basis for composing diets and lifestyle, thus shaping its quality and the consumer’s well-being [25].

The growing concern about the impact of dietary choices on the condition of the environment [21,26,27,28], food safety [29], and an increase in incidence rates of civilization diseases resulting from the poor-quality diets, [30,31], indicate the need for recognizing the importance of diets in the context of broadly defined life quality identified with well-being. An increasing number of researchers dealing with the issue of well-being recognize the important role of dietary behaviours [2,13,32,33,34,35]. In 2009, the concept of FWB appeared in scientific discourse [4]. It led to the reorientation in the approach to food consumption processes, with emphasis put on the need for developing proper attitudes and behaviours, focused not only on health, but also on overall well-being. As a result, food consumption ceased to be perceived only as the provision of nutrients, but the psychological [6,14,36,37,38,39]; and social [29,40,41] implications of this process for an individual were also recognized. The holistic approach to the role of food and dietary practices in human life takes into account the social and psychological dimension of consumption, abandoning the orientation which stems from the biomedical model of health [42]. Research carried out in this field shows that food consumption and its impact on well-being are related to the perception of health condition, a sense of pleasure and emotional aspects [8,13,17].

According to some authors, well-being may both be the effect of consumption and determine purchasing patterns [43]. Research into the influence of a diet on a person’s well-being brings ambiguous results. Oke et al. [44] point out that ethical food consumption is motivated by personal health and well-being. Forestell and Nezlek [45] have found that vegetarians and semi-vegetarians are more neurotic and depressed than omnivores. As the findings of the study by Pfeiler and Egloff [46] show, a vegetarian diet does not affect SWB whatsoever. In turn, Seconda et al. [47] found that the consumption of organic food impacts life satisfaction, while Apaolaza et al. [48] argue that it may be related to the label effect rather than the actual improvement of well-being.

The findings of the previously mentioned analyses indicate that people’s well-being is related to the categories and quality of the food they consume. Those studies were conducted mainly with reference to the influence of the frequency of organic food consumption on well-being, i.e., [48,49,50]. They have proven that the more frequently organic food is consumed, the higher the perceived well-being level is (in the physical, psychological, and social sphere). This concerns, among others, vegetarians, vegans and people on a gluten-free diet. What is more, consumers choosing organic food are also more sensitive to the health benefits of a diet. Springfield et al. [51] point to the quality of a diet and its impact on consumers’ health, while Pandya [52] argues that food allergies and elimination diets have a negative influence on the quality of life. On the other hand, Norwood et al. [53] indicate that the necessity of maintaining dietary discipline (even restrictive) does not have to entail lowered psychological well-being. In our research we also deal with diets with special regime, and we name them “exclusionary diets”. By this term we mean diets excluding or reducing specific food products ingredients, such as animal source components (dairy, meat), gluten, salt, fat, or sugar. We decided to take this approach as there is no universal expression for this type of diet, and elimination or reducing diets are medical expressions with specific definitions. Thus, our contribution to the research is the inclusion of other diets and categories of food consumed as a correlate of the level of FWB and SWB perceived by consumers. In this paper, we pose a question whether the type of food consumed, i.e., a specific diet, is related to the level of SWB and FWB.

We also analysed issues of health motivation to the consumption of specific food categories and the relationship between food consumed and the level of well-being. The results of studies carried out by Ares, de Saldamando, Giménez, and Deliza’s [13] and Ares et al. [8] concerning the evaluation of well-being with regard to specific products or food categories show that the influence of a diet on well-being refers to issues connected with perceived physical health, pleasure and emotional aspects. Moreover, motivation to the consumption of organic food rises under the influence of attitudes toward health-related and psychological consequences of consuming organic foods [50]. In turn, Apaolaza et al. [48] have proven that health-concerned consumers may enhance their well-being level by including organic foods in their diet. Thus, we decided to check whether such relationships occur in the case of other diets, too. Since the choice of a specific diet is dependent on consumers’ various motivations, we wanted to check if this issue is also related to the level of FWB and SWB. We were particularly interested whether people driven by single motives, such as the health, pleasure or social dimension of food declared the lower/higher level of SWB and FWB than those motivated by a larger number of factors.

Only a few researchers have adopted a holistic approach to the analyses of consumers’ food patterns, preferring the identification of the influence of specific diets or changes in them on well-being [54]. What is more, the existing body of literature provides a large number of examples of diets which are difficult to classify [55]. In our research we aim to identify consumers’ well-being from the perspective of following a specific diet (organic, ethical, convenient, sugar-free, gluten-free, and fat-reduced food), and to discover whether the particular dietary patterns are related with increased FWB and SWB. Additionally, we examine relations between FWB and SWB among Polish consumers, referring the results to the findings of international studies.

Based on the presented literature review we formulated the following hypotheses in our study:

**Hypothesis** **1** **(H1).** *Regular consumers of organic food, or food that improves mood, or specific exclusionary diets declare higher levels of SWB and FWB than people who do not follow such diets or follow them occasionally.*

**Hypothesis** **2** **(H2).** *Consumers who regularly eat convenient food declare the lower level of SWB and FWB than people who do not eat it at all or eat it occasionally.*

**Hypothesis** **3** **(H3).** *People who pay attention to the health, pleasure and social dimension of food while choosing a specific diet declare the higher level of SWB and FWB than those selecting a dietary pattern based on only one motive.*

## 2. Materials and Methods

### 2.1. Procedure and Participants

The study was conducted using an Internet research panel of the research company Biostat. The panel covers a nationwide database of consumers and allows for the sample to be selected according to specific characteristics, while ensuring the representativeness of the Polish population. The panelists were invited to the survey by e-mail generated by the system with a button directing to the questionnaire or the push notification in mobile application. The respondents completed the questionnaire using a web application or a mobile application. The needed sample size was calculated. The first 1067 respondents who completed the questionnaire in 100% and fit into the sample distribution assumed in the study were eligible for the study. The survey was conducted in agreement with the Declaration of Helsinki. The data was encrypted and is stored according to the General Data Protection Regulation.

1067 respondents took part in the study. The sample was representative for Polish citizens in the age of 18–54 divided according to gender, age, and place of residence (confidence interval 95 per cent, maximum random error 3%). Table 1 shows the sample characteristics.

### 2.2. Instruments

In order to verify our hypotheses, we applied scales which measure both SWB and FWB, as well as scales that allow for identifying consumers’ attitudes to food:

- The Satisfaction With Life Scale (SWLS) developed by Diener et al. [12] is a five-item scale that measures global cognitive judgement of SWB on a seven-point scale. After summing up, individual scores produce the overall score reflecting the level of life satisfaction. The possible range of scores is 5–35—the higher the score is, the higher the level of satisfaction with life is. This scale showed good internal consistency with Cronbach’s α between 0.79 and 0.89 [11,12,56,57,58]. Studies conducted in Poland reported high internal consistency of the scale (0.81) in its Polish version [59]. In our study, the Cronbach’s alpha of the SWL scale was 0.89.
- The scale measuring the level of satisfaction with life related to food [2]—Satisfaction with Food-related Life Scale (SWFL). SWFL scale consists of five items concerning different aspects of food consumption. The respondents must indicate their degree of agreement with the statements using a six-level Likert scale. Scores obtained on this scale range from 5 to 35 and the higher the score is the higher FWB is. The Polish version of the scale was used in the original study conducted by [2]. This scale showed good internal consistency with Cronbach’s α between 0.79 and 0.90 in studies conducted in European countries [2], South American country Chile [60] and in China [11]. In our study, SWFL’s Cronbach’s α was 0.86 presenting an adequate level of internal consistency.
- HTAS (Health Taste Attitude Scales) developed by Roininen, Lähteenmäki and Tuorila [61] measures the importance of health and taste aspects of a diet in the food choice process. We applied the general health interest subscale (hHTAS) with eight items and the pleasure sub-scale (pHTAS) with six items. All responses were measured on the seven-level Likert scale. The Polish version of HTAS scale was prepared by [62]. In our study, hHTAS’s Cronbach’s α was 0.74 presenting an adequate level of internal consistency. pHTAS’s Cronbach’s α was 0.55, therefore, in order to increase the scale’s consistency, we removed the three items of low contribution to the Cronbach α. The three-item pHTAS scale was related to taking pleasure from the taste of food and showed a good internal consistency with Cronbach’s α 0.73.
- Social dimension of food meaning (SMFL) taken from the scale developed by Arbit, Ruby and Rozin [63], measuring the meaning of food in life (MFL). In this scale, we used four statements evaluated on the seven-level Likert scale. The items of SMFL scale were translated into Polish and back translated to ensure a valid translation. Afterwards the Polish version of the scale was consulted with three independent scientists, experts in the field of food consumption research. The Cronbach’s alpha of the SMFL scale in this study was 0.84, what proves about high internal consistency of the scale.

All scales can be found in Appendix A.

The questionnaire included other questions related to the frequency of consumption of various food categories as well. Finally, questions for socio-demographic classification were included (gender, age, financial status, level of education, weight, growth, the assessment of overall health condition).

### 2.3. Data Analysis 

In the data analysis process we applied descriptive techniques: measures of location, diversification, asymmetry and concentration, which were used for the preliminary analyses and description of the examined sample. At this stage, we also used the χ^2^ test. For the comparison of the mean scores of SWB and FWB in the specific groups of respondents we used one-way analysis of variance (ANOVA) and partial eta squared as a measure of effect. For the data which meet the assumption of the homogeneity of variance (based on Levene’s test), we applied post-hoc Hochberg’s GT2 test, while with reference to data which do not meet this assumption, we applied the Games–Howell test, because both of them can be used for non-equipotent groups. By using one-way ANOVA we were able to show the influence of independent variables on the dependent ones, but no relationships between independent variables were taken into account. To verify whether people who pay attention to the health, pleasure and social dimension of food declare higher levels of SWB and FWB than people who only care about one motive, we used independent-samples t-Test and Cohen’s d. Pearson’s correlation analysis was used to investigate the relationship between SWB and FWB. The results were analysed using the SPSS Statistics vs. 26.0. programme for Windows.

## 3. Results

The first objective of this study was to analyse the existence of a positive relation between Satisfaction with Life (SWB) and Satisfaction with Food-related Life (FWB) among Polish consumers. To this end, we calculated satisfaction levels for all respondents, with the application of SWFL and SWLS scales (Table 2).

In this research stage, we verified the first two hypotheses. As the first step, we analysed the frequency of consumption of the specific food categories, and then calculated the levels of SWB and FWB in the particular groups of respondents, who differ in frequency of the use of individual diets and compared mean scores with the application of one-way ANOVA along with respective post-hoc tests (Table 3).

Results of the verification of detailed hypotheses concerning the influence of diets on the level of SWB and FWB are as follows:

- Differences in the level of SWB and FWB among people declaring different frequencies of the consumption of organic food are statistically significant on the level *p* < 0.001. The regular consumers of organic food declare the higher level of SWB and FWB than the respondents who do not follow such a diet or follow it occasionally.
- The regular consumers of the food which improves mood showed the higher level of FWB than people who eat it irregularly or do not eat it at all. Differences between the mean scores of FWB observed in these groups were statistically significant on the level *p* < 0.001. Differences in mean scores (SWB) were not statistically significant.
- With regard to the relationship between the frequency of exclusionary diets and the level of SWB it can be concluded that the regular followers of some exclusionary diets (low-salt, low-sugar, gluten-free) have the higher level of SWB than those who do not follow them at all. Differences between the levels of SWB in the groups of regular and occasional consumers were statistically significant only in the case of the consumption of low sugar products consumption.
- The regular users of the following diets: low-fat, low-salt and low-sugar showed the higher level of FWB than those who do not follow them at all or use them occasionally. In the case of vegans, significant differences were observed only between the groups of regular consumers and non-consumers. Differences in the levels of FWB concerning the frequency of consumption of vegetarian, lactose-free and gluten-free food were not statistically significant. It can therefore be concluded that the FWB level was higher for people regularly using some exclusionary diets.
- For the SWB, H1 was supported by the data only for organic and low-sugar diets. Concerning FWB, H1 was verified for the low-salt diet, low-fat diet, and the food improving mood food as well. It can thus be confirmed that more dietary patterns are related to the level of FWB than to the level of SWB. In both aspects, the consumption of organic food has a positive impact, while the influence of exclusionary diets is ambiguous. That is why, in further analysis we analysed how the combination of a few exclusionary diets correlates with SWB and FWB.
- The regular consumers of convenient food exhibited the lower level of SWB than the respondents who eat such food only occasionally. With reference to FWB, the examined relationship was not statistically significant at all. The H2 was not supported by the data.

Seeking further relationships between dietary patterns and the level of well-being, we undertook discovering the relationship between the number of diets excluding or reducing specific ingredients of food products followed and the levels of FWB and SWB. The highest levels of FWB and SWB were observed in the groups of people who regularly follow at least four exclusionary diets, while the lowest were among the consumers who do not regularly use any diet and those who follow only one diet. The ANOVA analysis and Hochberg GT2 post hoc test revealed that people following a variety of diets differed statistically significantly in terms of the declared level of SWB and FWB: F_SWB_(3.1063) = 4.491; *p* < 0.01; ŋ^2^ = 0.013 and F_FWB_(3.1063) = 12.754, *p* < 0.001, ŋ^2^ = 0.035. Table 4 presents the comparison of homogeneous groups of SWB and FWB means. The means in each distinguished group (column) do not significantly differ from each other. Thus, with regard to SWB, people who do not follow any exclusionary diet and those who pursue at least four such diets are statistically significantly different. In the case of FWB, three homogeneous groups were established: people who do not follow such a diet, people who pursue one to three exclusionary diets and those who use at least four diets.

So far it has been shown that the regular consumption of organic food is associated with increased SWB and FWB. Other diets had similar effects, although it was difficult to find any regularities (e.g., diet restrictiveness), which would allow us to make some general statements concerning exclusionary diets. That is why we decided to analyse consumers’ attitudes affecting the choice of a diet.

In order to verify hypothesis H3, we conducted Student’s t-test for independent samples. The groups were distinguished on the basis of quartiles. In the analysis, we took into consideration people belonging to the highest quartiles according to hHTAS, reduced pHTAS and SMFL. As regards SWB, the analysis showed that the average level of SWB of those who pay attention to the health, pleasure and social dimension of food is statistically significantly higher (M = 25.00; SD = 6.17) than the SWB level of people who only care about the health issues (M = 20.96; SD = 6.22; t(203) = 4.40; *p* < 0.001, d = 0.650) or pleasure (M = 21.56; SD = 6.77; t(150) = 3.625, *p* < 0.001, d = 0.591) or social dimension of food (M = 22.34; SD = 6.68; t(163) = 2.601, *p* = 0.01, d = 0.411). The same relationships were observed in the case of FWB. The average level of FWB of those who pay attention to the health, pleasure and social dimension of food is statistically significantly higher (M = 24.93; SD = 3.73) than the FWB level of people who only care about the health issues (M = 21.21; SD = 3.97; t(203) = 6.46; *p* < 0.001, d = 0.954) or pleasure (M = 21.66; SD = 4.51; t(150) = 4.796, *p* < 0.001, d = 0.781) or social dimension of food (M = 21.31; SD = 4.75; t(163) = 5.256, *p* < 0.001, d = 0.830 (Table 5). This means that hypothesis H3 was positively verified.

Effect size (Cohen’s d) in the case of health was higher than for the other single motives, which means that adding the remaining motives to health increased SWB and FWB more than adding two motives to pleasure or social dimension. This influence was more significant with regard to FWB than SWB.

## 4. Discussion

In our study, we examined whether there is a link between consumption and motivations to follow specific diets (organic, vegetarian, vegan, convenient, lactose-free, gluten-free, salt-reduced, sugar-reduced and fat-reduced food) and the levels of FWB and SWB.

Our research results show that there is a strong relation between life satisfaction measured on the SWLS scale and satisfaction connected with the area of food consumption, measured on the SWFL scale (r-Pearson correlation between SWB and FWB was r = 0.59 and was statistically significant on the level *p* < 0.01 (both ways). The correlation between SWB and FWB in the present study is similar to those obtained in China [11], which was 0.58, in Chile: 0.53 [60], and higher than in Ecuador: 0.39 [64] and in European countries [2], which was 0.36. It should be pointed out that not only the overall scores of SWLS and SWFL, but also all statements from both scales are correlated. This means that nutrition and satisfaction or dissatisfaction with food consumption are associated with the broadly defined well-being. These findings are in line with the current state of knowledge and are consistent with the results of studies of, among others [11,60,64,65].

A novelty in our study is that we referred the levels of SWB and FWB to the use of different diets defined as dietary patterns. A positive relationship occurs mainly with regard to the consumption of organic food, which confirms the findings of previous studies in this area e.g., [48,50]. The regular consumption of organic food does not mean eliminating some product categories, but it involves the selection of food with additional quality benefits. That is why this positive relationship between the frequency of consumption of high-quality food and the higher perceived satisfaction with life is easy to understand. What is striking is the observed dependency between adopting exclusionary diets and well-being. The abandonment of specific food product categories may increase a sense of discomfort after all. It turns out, however, that the use of diets involving different kinds of eliminations or reductions does not have to make a consumer feel limited in their food choice, thus be less satisfied with life. In many cases, the opposite is the case. The regular use of vegan, low-salt and low-sugar diets is related to high SWB, while, in addition to these diets, the frequent consumption of low-fat products correlates with the high level of FWB. The influence of the regular use of specific exclusionary diets on the levels of FWB and SWB is not homogeneous. One of the reasons could be the trigger to adopting such a diet—whether it was because a consumer wanted it or whether he or she was forced to it because of his or her physical condition.

When analysing consumers’ dietary choices, the motivation to make them is an important area that should be taken into account. An individual’s physical condition and health, but also the pleasure and social dimension of food consumption are a few among many other motives. Therefore, these motivations for choosing different diets are key. A specific diet can be chosen because of the same or of the various motives. However, in our research, it turned out that single motives are less important for the levels of FWB and SWB than their combination. We proved in our research that people who pay attention to the health, pleasure and social dimension of food while choosing a specific diet, declared the higher level of SWB and FWB than those selecting a dietary pattern based on only one motive. These results should be used to encourage consumers to adopt specific diets. It is only the combination of all these areas in the promotion of dietary patterns that may lead to a permanent change in people’s eating habits. Therefore, we have to educate consumers to raise awareness that a diet can be tasty and that its observance can become a positive element of social relations, because, apart from pro-health aspects, it is equally important to take pleasure from having meals together and have the support of a family. Like other studies, ours also has some limitations, which, at the same time, offer some clues regarding the directions of further research. We did not perform the evaluation of the quality of diets used by the respondents, but we used their declarations concerning the diet they observed. In future research, more precise tools may be applied, such as the Stanford Wellness Living Laboratory (WELL) or Food Frequency Questionnaire (FFQ). They will make it possible to assess different diets more accurately [51]. Moreover, we compared the levels of SWB and FWB between the respondents following or not following a particular diet. Because of the design of our research, we could not compare the levels of SWB and FWB between the groups following different dietary patterns, as these groups were not distinct. Another limitation is that because we removed the three items of low contribution to the Cronbach α from the original pHTAS scale to increase the scale’s consistency, our results are less comparable with other studies using the original version.

In our study, we examined the orientation on health, pleasure and socialization as a motivation to follow a diet, so the number of the analysed motives was limited by including a larger number of motives, we would be able to distinguish, among others, people who are on a diet by choice (beliefs, fashion) from those who follow them because of allergies or food intolerance. This would contribute to the broadening of knowledge connected with FWB and SWB. That is why further research is needed in the area of consumers’ motivation to adopt a particular dietary pattern, with special attention given to the complexity and interdependence of motives. Knowing the reasons behind the nutritional choices which contribute to increasing the level of life satisfaction, we can be more effective in adjusting argumentation encouraging consumers to make dietary decisions that would benefit them. If we only appeal to pro-health aspects, we will not achieve the expected results. We also need to pay attention to the social dimension and pleasure.

In order to identify changes of motivation and behaviour in time, it will also be worth introducing longitudinal studies [66]. For the sake of deepening knowledge concerning dietary patterns and the resulting well-being, future research should take into consideration the situational and cultural context in which food and diets are chosen. The effective change of a diet is determined by the support of a family, friends, schoolmates and of the whole society. This positive change can be also encouraged with the help of new technology-based solutions as well, such as mobile applications. Given the above, future studies should also explain the role of the different sources of support in fostering dietary habits characteristic of specific diets.

## 5. Conclusions

What is the main contribution of this paper is that it provides empirical evidence that the relationship between dietary patterns and food consumption motivations and consumers’ food and subjective well-being exists? The dietary choices we make, and the reasons behind them are therefore important correlates of our well-being. Previous studies of the influence of food consumption on SWB and FWB to a limited extent took into account the motivation to adopt a diet as a factor differentiating an individual’s SWB and FWB levels. Our paper fills this gap and provides conclusions concerning the shaping of dietary patterns desired from the point of view of both the individual and the society.

Dieticians usually recommend various food choices that contribute to the better health of the individual. On the other hand, consumers are guided in their decisions by various motivations related to, inter alia the pleasure of eating, the social dimension of consumption, but also the desire to decrease the environmental impact of food consumption practices. Our research shows that coexisting differentiated motivations show a positive relationship with food and subjective well-being. A consumer engaged in various nutritional goals, using a wider range of diets, experiences the higher level of satisfaction with the quality of his or her life.

Knowledge concerning people’s motivation to follow specific diets, as well as the occurrence of the relationships between food consumption and an individual’s overall well-being may be used for creating communication to support proper dietary decisions. According to our research, food products’ communication based on health values and benefits may not bring optimal results in terms of the growth of well-being. It is necessary to take into consideration the psychological and social aspects of SBW [50]. The findings of our study shed light on the important aspects that should be taken into account when communicating these issues to society. This communication should include not only health benefits and effects, but also references to the social aspects of consumption and happiness because consumers are motivated not only by their concern about health, but also by pleasure and the social dimension of consumption.

## Figures and Tables

**Table 1:** The profile of respondents.

Variables	Frequency (%)
Total	1067 (100.0)
Gender	
Female	504 (47.2)
Male	563 (52.8)
Age	
18–24 years old	186 (17.4)
25–34 years old	351 (32.9)
35–44 years old	327 (30.6)
45–54 years old	203 (19.0)
Level of education	
Primary	30 (2.8)
basic vocational	115 (10.8)
Secondary	474 (44.4)
Higher	448 (42.0)
Place of residence	
Village	425 (39.8)
city up to 20,000 inhabitants	139 (13.0)
city from 20,000 to 100,000 inhabitants	205 (19.2)
city from 100,000 to 200,000 inhabitants	85 (8.0)
city from 200,000 to 500,000 inhabitants	90 (8.4)
city over 500,000 inhabitants	123 (11.5)
The assessment of the financial situation	
very bad or bad	66 (6.2)
Average	489 (45.8)
Good	458 (42.9)
very good	54 (5.1)
The assessment of the overall health condition	
definitely bad or rather bad	88 (8.2)
neither poor nor good	197 (18.5)
quite good	627 (58.8)
definitely good	155 (14.5)
BMI	
Underweight	37 (3.5)
normal weight	555 (52.0)
overweight	344 (32.2)
Obesity	131 (12.3)
Number of dietary patterns followed	
None	191 (17.9)
One	177 (16.6)
Two	241 (22.6)
Three	136 (12.7)
Four	127 (11.9)
five or more	195 (18.3)

**Table 2:** Basic data concerning the level of SWB and FWB.

Measure		SWB	FWB
*N*		1067	1067
Average		21.14	20.59
Median		22.00	21.00
Mode		25.00	22.00
Standard deviation		6.45	4.94
Minimum		5.00	5.00
Maximum		35.00	30.00
Percentile	25	17.00	17.00
	50	21.00	22.00
	75	24.00	26.00

SWB: Satisfaction with Life; FWB: Satisfaction with Food-related Life.

**Table 3:** Frequency of consumption different food category and the level of FWB and SWB.

Dietary Pattern	Regularity		Frequency (%)	Mean SWB	Mean FWB
Organic *n* = 979	Regularly		301 (30.7)	22.82 ^a^	22.09 ^a^
	sometimes		612 (62.5)	20.99 ^b^	20.45 ^b^
	Never		66 (6.74)	19.17 ^b^	18.45 ^c^
ANOVA; post hoc; ŋ^2^		F_SWB_(2, 976) = 13.471, *p* < 0.001, GT2H; ŋ^2^ = 0.027; F_FWB_(2, 976) = 21.054, *p* < 0.001, GT2H; ŋ^2^ = 0.041			
Vegetarian *n* = 967	Regularly		190 (19.6)	21.52	21.42
	sometimes		503 (52.0)	21.57	20.65
	Never		274 (28.3)	20.84	20.60
ANOVA; post hoc		F_SWB_(2, 964) = 1.267; *p* > 0.05; F_FWB_(2, 964) = 2.010; *p* > 0.05			
Vegan *n* = 929	Regularly		85 (9.1)	21.23 ^a,b^	21.80 ^a^
	sometimes		422 (45.4)	22.16 ^a^	20.95 ^a,b^
	Never		422 (45.4)	20.81 ^b^	20.43 ^b^
ANOVA; post hoc; ŋ^2^		F_SWB_(2, 926) = 4.872; *p* < 0.01; GT2H; ŋ^2^ = 0.01; F_FWB_(2, 926) = 3.297; *p* < 0.05; GH			
Low fat diet *n* = 924	Regularly		324 (35.1)	21.95	21.87 ^a^
	sometimes		521 (56.4)	21.24	20.23 ^b^
	Never		79 (8.5)	20.66	19.42 ^b^
ANOVA; post hoc; ŋ^2^		F_SWB_(2, 921) = 1.958; *p* > 0.05; F_FWB_(2, 921) = 15.054; *p* < 0.001; GT2H; ŋ^2^ = 0.031			
Gluten-free *n* = 866	Regularly		87 (10.0)	22.54 ^a^	21.46
	sometimes		396 (45.7)	21.75 ^a,b^	20.74
	Never		383 (44.2)	20.75 ^b^	20.64
ANOVA; post hoc		F_SWB_(2, 863) = 4.034; *p* < 0.05; GT2H; F_FWB_ (2, 863) = 1.025; *p* > 0.05			
Lactose-free *n* = 856	Regularly		88 (10.3)	21.83	21.22
	sometimes		347 (40.5)	21.52	20.58
	Never		421 (49.2)	21.22	20.76
ANOVA; post hoc		F_SWB_(2, 853) = 0.450; *p* > 0.05; F_FWB_ (2,853) = 0.626; *p* > 0.05			
Low-salt diet *n* = 882	Regularly		276 (31.3)	22.12 ^a^	21.75 ^a^
	sometimes		464 (52.6)	21.35 ^a,b^	20.45 ^b^
	Never		142 (16.1)	20.16 ^b^	19.82 ^b^
ANOVA; post hoc; ŋ^2^		F_SWB_(2, 879) = 4.587; *p* < 0.05; GT2H; ŋ^2^ = 0.01; F_FWB_ (2, 879) = 9.436; *p* < 0.001; GH; ŋ^2^ = 0.021			
Low-sugar diet *n* = 930	Regularly		378 (40.6)	22.17 ^a^	21.51 ^a^
	sometimes		436 (46.9)	21.01 ^b^	20.23 ^b^
	Never		116 (12.5)	19.49 ^b^	19.96 ^b^
ANOVA; post hoc; ŋ^2^		F_SWB_(2, 927) = 8.930; *p* < 0.001; GT2H; ŋ^2^ = 0.019; F_FWB_ (2, 927) = 8.913; *p* < 0.001, GT2H; ŋ^2^ = 0.019			
Improving mood *n* = 965	Regularly		505 (52.3)	21.37	21.34 ^a^
	sometimes		414 (42.9)	20.94	19.91 ^b^
	Never		46 (4.8)	20.83	19.54 ^b^
ANOVA; post hoc; ŋ^2^		F_SWB_(2, 962) = 0.593; *p* > 0.05; F_FWB_ (2, 962) = 11.132; *p* < 0.001; GT2H; ŋ^2^ = 0.023			
Convenient *n* = 1018	Regularly		506 (49.7)	20.37 ^a^	20.35
	sometimes		481 (47.2)	21.89 ^b^	20.82
	Never		31 (3.0)	21.58 ^ab^	20.87
ANOVA; post hoc; ŋ^2^		F_SWB_(2,1015) = 6.978; *p* < 0.01;GH; ŋ^2^ = 0.02; F_FWB_ (2, 1015) = 1.144; *p* > 0.05			

Note: The analyses and the table took into consideration ŋ^2^, which explains at least one per cent of the variability of the SWB and FWB. Mean values with different superscripts differ significantly (GH: Games-Howell test; *p* < 0.05, GT2H: GT2 Hochberg test; *p* < 0.05). The means for a given dietary pattern differ statistically significantly only when marked with different letters (e.g., FWB mean for the regular followers of a vegan diet (M = 21.80 ^a^) is statistically different from the mean for people who never pursue this diet (M = 20.43 ^b^). The mean for people who sometimes observe this diet does not differ statistically significantly from the other groups (M = 20.95 ^a,b^).

**Table 4:** SWB and FWB levels in the groups of respondents distinguished according to the number of diets followed.

Number of Exclusionary Diets	*n*	Mean SWB		Mean FWB		
		Subset for α = 0.05		Subset for α = 0.05		
		1	2	1	2	3
Four and more diets	112	22.36		22.49		
Two or three diets	287	21.70	21.70		21.12	
One diet	186	21.52	21.52		20.97	
No diets	482		20.39			19.69
Sig.		0.219	0.714	1.00	1.00	1.00

Note: Subset for α = 0.05 means that subgroups between which means differ with statistical significance α = 0.05 were marked. Sig. refers to the significance of differences between means inside the column.

**Table 5:** The comparison of the average SWB and FWB levels of people driven by three motives and those motivated exclusively by the health issues or pleasure or social dimension of food.

Type of Well-Being	Motives	M	SD
SWB	Health + Pleasure + Social dimension of food	25.00	6.17
	Health	20.96	6.22
	Pleasure	21.56	6.77
	Social dimension of food	22.34	6.68
FWB	Health + Pleasure + Social dimension of food	24.93	3.73
	Health	21.21	3.97
	Pleasure	21.66	4.51
	Social dimension of food	21.31	4.75

## Data Availability

The datasets analysed during the current study are available from the corresponding author on a request.

## Appendix A

**A. Satisfaction with Life Scale (SWLS),** 7-point Likert Scale (1: disagree completely, 7: agree completely) [12], Polish version [59]
  1. In most ways my life is close to my ideal.
  2. The conditions of my life are excellent.
  3. I am satisfied with my life.
  4. So far I have gotten the important things I want in life.
  5. If I could live my life over, I would change almost nothing.
**B. Satisfaction with Food-related Life Scale (SWFL),** 6-point Likert scale (1: disagree completely, 6: agree completely) Polish version used in original research of [2]
  1. Food and meals are positive elements
  2. I am generally pleased with my food
  3. My life in relation to food and meals is close to ideal
  4. With regard to food, the conditions of my life are excellent
  5. Food and meals give me satisfaction in daily life
**C. Health Taste Attitude Scales—health (hHTAS)** 7-point Likert (1: disagree completely, 7: agree completely) [61]; Polish version [62]
  1. The healthiness of food has little impact on my food choices.
  2. I am very particular about the healthiness of food I eat.
  3. I eat what I like and I do not worry much about the healthiness of food.
  4. It is important for me that my diet is low in fat.
  5. I always follow a healthy and balanced diet.
  6. It is important for me that my daily diet contains a lot of vitamins and minerals.
  7. The healthiness of snacks makes no difference to me.
  8. I do not avoid foods, even if they may raise my cholesterol.
**D. Pleasure Health Taste Attitude Scales (pHTAS)**, 7-point Likert Scale (1: strongly disagree, 7: strongly agree) scale [61]; Polish version [62]
  1. I do not believe that food should always be source of pleasure.
  2. The appearance of food makes no difference to me
  3. When I eat, I concentrate on enjoying the taste of food.
  4. It is important for me to eat delicious food on weekdays as well as weekends.
  5. An essential part of my weekend is eating delicious food.
  6. I finish my meal even when I do not like.
**E. Social Dimension of Food Meaning (SMFL),** 7-point Likert Scale (1: disagree completely, 7: agree completely) [63]; Polish version: own translation)
  1. Sharing food with others makes me feel closer to them
  2. When I eat food I feel connected with the people I am eating with
  3. Food is closely tied to my relationships with others
  4. Making food for others is a main way I show care for them
